# Students’ learning in clinical practice – a scoping review of characteristics of research in the Nordic countries

**DOI:** 10.1080/10872981.2023.2279347

**Published:** 2023-11-18

**Authors:** Charlotte Silén, Susanne Kalén, Pernilla Lundh, Janet Mattson, Katri Manninen

**Affiliations:** a Department of Learning, Informatics, Management and Ethics, Karolinska Institutet, Stockholm, Sweden; b Department of Clinical Science and Education, Södersjukhuset, Karolinska Institutet, Stockholm, Sweden; c Department of Education, Health and Medical Care Administration, Region Stockholm, Stockholm, Sweden; d Children’s Perioperative Medicine and Intensive Care, Karolinska University Hospital, Stockholm, Sweden; e Department of Infectious Diseases, Karolinska University Hospital, Stockholm, Sweden

**Keywords:** Clinical practice, health care students, Nordic context, scoping review, student learning

## Abstract

**Rationale:**

The complex nature of student learning in clinical practice calls for a comprehensive pedagogical framework on how to create optimal learning affordances.

**Purpose:**

The purpose of this study was to describe characteristics of conducted research regarding investigated research questions, distribution of different health care student groups, and employed methodological approaches.

**Methods:**

A scoping review was chosen to capture the multifaceted characteristics in the field of learning in clinical practice. Funded local projects were analysed to provide significant core concepts for the literature search. A systematic search and review of articles published 2000–2019 in the Nordic countries was conducted according to PRISMA- ScR (23). The search was made in Medline (OVID), SveMed+ and CINAHL and resulted in 3126 articles. After screening of the titles and abstracts 988 articles were included for further review. The abstracts of all these articles were reviewed against established inclusion and exclusion criteria and 391 articles were included. Characteristics of purposes and research questions were analysed with a qualitative content approach resulting in identified subject areas including significant categories. Health care student groups and methodological approaches were also identified.

**Results:**

Subjects predominating the research were *organisation of clinical practice, supervision*, and *students’ experience* followed by *interprofessional learning* and *learning environment*. *Co-operation, university-clinical setting*, and *patients’ role* were investigated to a small extent. Sparsely occurring subjects were also *specific learning outcomes* and *evidence-based knowledge*. Nursing students were involved in 74% of the studies, medical students in 20%, and other professions around 8%. Qualitative approaches were most common.

**Conclusion:**

Health care students’ learning in clinical practice has been researched to a large extent within the Nordic countries and important subject areas are well represented. The research displays a great potential to extract and describe factors to create a pedagogical framework with significant meaning to support students’ learning.

## Introduction

Clinical practice as a part of undergraduate and postgraduate health care education is recognised as very important for students’ learning and professional development. There are many factors that affect student learning in clinical practice. The clinical learning environment is influenced by issues such as the supervisors’ interest and knowledge, space and time dedicated for students at the clinical placement and the character of the caring and patient encounters [1–3]. Intentions to create optimal prerequisites for learning when students take part in and interact with their future professional practice is certainly judged as significant to high quality health education [4,5]. Studies investigating different issues can guide educational strategies. Considering the complex nature of clinical practice, we would argue that there is a need of a comprehensive evidence basis on how to create prerequisites for health care students’ optimal learning in clinical practice. A description and analysis of issues researched on a large scale could serve as a well-informed basis to identify fruitful educational strategies. To inspire future research, it is also important to highlight significant issues less researched.

There are several areas of interest to highlight regarding students’ learning in clinical practice. From a socio-cultural and constructivist perspective, learning comes about through active processing of information and a construction of understanding, skills, and attitudes. These processes involve cognitive, emotional, and social aspects as well as practical actions and interactions between the dimensions content, incentives, and environment [6–8]. The intention is for the students to develop a profession and it implies learning health care knowledge and skills, but also values and attitudes [9]. This means that students’ learning in clinical practice is a complex endeavour, and there are many aspects involved that impact students’ learning processes [1–3]. The clinical environment offers unique pedagogical encounters and rich learning situations comprising interactions with patients and staff, skills training and activities requiring application of and reflection on gained theoretical knowledge [3,9]. Several studies verify that the experiences of authenticity and opportunities to apply theoretical knowledge in their future profession facilitates students’ understanding of it, boosting their feeling of meaningfulness [5,10,11]. Experiences of authenticity in the clinical environment play an important role in the occurrence of relevance and meaningfulness [12–14]. However, the students’ education will depend on structures and learning resources being recognised and adapted to students’ needs for prerequisites and support if they are to be enabled to benefit effectively from their clinical practice [15]. One major issue is that students are only temporary visitors in contexts where the delivery of health care is the primary task and supporting students’ learning will usually come second [16]. Supervisors’ behaviour and pedagogical knowledge, their relationship to students and the organisation of supervision have proven to be very important for students’ ability to achieve intended learning objectives in clinical practice [4,17–21]. However, the socio-cultural environment including interactions with staff, patients, and other health care students is likely to affect how students experience and can benefit from clinical practice for their learning [3,8,11,22]. The relationships and interactions between health care actors and higher education institutions form basic prerequisites for how clinical practice is organised and affecting the students learning.

Scoping reviews are pointed out as useful to answer broad questions, identify conducted research as well as knowledge gaps and to provide characteristics signify a certain area (PRISMA Extension for Scoping Reviews [23–25]. The presentation above of the variety of factors affecting student learning in clinical practice points out the usefulness of a scoping review to map existing research regarding this complexity. The purpose of this review is thus to provide an overview of the characteristics of conducted research, as well as existing evidence and knowledge gaps, concerning students’ learning in clinical practice in terms of:

- the kind of research questions that are examined
- the distribution of research related to different health care student groups
- methodological approaches that have been used.

## Material and methods

The method of the scoping review followed the guidelines outlined in PRISMA-ScR [23]. The implementation of the search strategy is presented below.

### Search strategy

To accomplish a solid basis for the search of pertinent articles to include in the scoping review the work began with an analysis of pedagogical research projects supported by grants provided by ALF, the Regional Agreement on Medical Training and Clinical Research between Region Stockholm and Karolinska Institutet. These projects were awarded funding to develop different aspects of clinical practice for all health care students. Since these projects were already assessed as valuable related to the development of clinical practice, they were judged suitable to contribute to an informed search strategy. All supported projects are official and registered at the local county council for health care. They were identified through requested information from the county council. The final reports of all granted projects between 2006–2017, *n* = 147, were read to identify any projects investigating students’ learning in clinical practice. Published articles within the included projects were collected, and articles within the scope of students’ learning were identified, *n* = 39. These articles were further analysed based on a study protocol (Appendix 1) developed and built on the methodological approach described by Joanna Briggs Institute [25]. According to the study protocol the research questions, existing theoretical frameworks, and described results of each article were mapped and analysed to identify any significant factors and concepts of importance key to understanding students’ learning in clinical practice. All authors were involved in an iterative process to analyse and discuss in particular the aims of the articles and the presented results. The following contextual factors came to the fore as important in influencing students’ learning: supervision, supervisors, relationships, teamwork, organisational prerequisites, learning/pedagogical activities, encounters with patients, health care professionals, intended learning outcomes, and forms of assessment and evaluation. Recurrent concepts investigated related to the students’ learning in clinical practice were meaningfulness, belongingness, authenticity, trust, and learning processes.

A comprehensive systematic search of literature was subsequently conducted with the assistance of an expert at the university library (Appendix 2, page 1, 2–3). The search was made in Medline (OVID), SveMed+ and CINAHL. Based on the contextual factors and concepts identified in the process described above keywords consistent with suitable MeSH terms were chosen. The MeSH terms used were student, placement, clinical clerkship, education, learning, preceptorship, mentoring, and supervisor. The literature search was conducted in March 2020 and was limited to articles published from 2000–2019 in the Nordic countries, including research articles published in English, Swedish, Danish, Norwegian, and Finnish. The reason for focusing on the Nordic countries was the opportunity to reflect similar conditions in terms of health care systems and education at a university level, as well as shared cultural values. Included were peer-reviewed articles, qualitative as well as quantitative, and both empirical studies and theoretical papers focusing on the keywords. After removing duplicates, the search resulted in 3126 articles (Appendix 2, flow diagram). They were uploaded in the management software Mendeley for further review and decision of inclusion. A list of inclusion and exclusion criteria was established to guide the review (Table 1).

**Table 1.** Criteria for inclusion and exclusion of articles in the review.

Criteria for inclusion	Criteria for exclusion
Student learning processes in clinical practice	Development of instruments e.g for assessment in clinical practice
Studies performed in the Nordic countries	Development of simulation devices
Undergraduate and postgraduate education	Education/courses directed to clinical supervisors but not clearly related to students learning
Interprofessional education in clinical practice	Descriptions and evaluations of curricula and courses
International exchanges if including Nordic students	Trade journals within health care
Nursing-, medical-, physiotherapy-, occupational- and biomedical analyst students	Psychology-, sociology-, dentist-, pharmacists-, biomedical scientists-, veterinary- students and residents
Peer reviewed articles	About clinical practice but not about health care students’ learning
	About student learning but not emphasizing clinical education
	Learning about a special subject e.g anatomy
	Studies about simulation
	Skills training not directly performed in clinical practice
	Theoretical/philosophical studies

First the titles and abstracts of the articles were screened by two of the authors to include eligible articles about: student learning processes in clinical practice, health care students and studies performed in the Nordic countries. The screening resulted in the inclusion of 988 articles (Appendix 2, flow diagram). In the next phase these articles were reviewed with all researchers involved. The abstracts of all articles were read and reviewed against the inclusion and exclusion criteria (Table 1). Related to the large number of studies the review and discussion about how to interpret inclusion and exclusion criteria was an iterative process. Finally, based on thorough consideration the review resulted in the inclusion of 391 studies (Appendix 2, flow diagram). All articles included in the review provided a title in English. 65 studies were published in Nordic languages. The included articles are presented in Supplement 1.

### Analysis

In the analysis and synthesis, a qualitative content analysis process was applied resulting in identified subject areas and manifest categories [26]. All included articles (*n* = 391) were divided in the research group for analysis. The researchers worked in three pairs to identify the focus and characteristics of the research conducted in the included studies. The purpose and research questions described in the articles were extracted and listed in Excel™ tables. Given the large number of studies included for the analysis this became an iterative process working in pairs followed by discussions in the whole research group. Ultimately this analysis resulted in an agreement to sort and describe the content of the articles in subject areas that mirrored common aspects and perspectives of students’ learning in clinical practice. Each subject area was further analysed to find characteristic features of described purposes and research questions. The content of the sorted articles within each subject area was compared to find similar characteristics of what had been researched. Similar characteristics were grouped and labelled as categories.

To further describe characteristics of the conducted research, the profession of the health care students and the methodological approach were identified in each article. The number of each group of identified health care students in the articles was summarised and related to the total sum of articles and subject areas. A similar procedure was applied concerning methodological approaches and the different approaches were summarised, described, and then related to the subject areas and categories.

## Results

The analysis of the 391 included studies is presented under three subheadings: *1. Subject areas; 2. Health care student groups;* and *3. Methodological approaches*. Due to the large number of included articles single studies are not referenced in the result presentation. However, to provide information about the included articles, the references listed in Supplement 1 are grouped in relation to identified subject areas.

### Subject areas

The analysis of the included articles resulted in the identification of nine significant subject areas about students’ learning in clinical practice. The nine subject areas are as follows: *organisation of the clinical practice, supervision, students’ experience, interprofessional learning, learning environment, specific learning outcomes, co-operation university-clinical setting, relation to evidence-based knowledge, and patients’ role*. The number of articles within each subject area varied between 6–100. Further analysis of the subject areas resulted in descriptive categories. The nine subject areas, the number of articles and the descriptive categories are displayed in Table 2 and further presented in the following order: from the subject area that contains the highest number of studies to the subject area with the lowest number.

**Table 2.** Descriptive categories and subject areas, number of articles in each subject area.

Subject areas	1. Organization of clinical practice *N* = 100	2. Supervision N = 80	3. Student’ experience N = 71	4. Interprofessional learning (IPL) *N* = 47	5. Learning environment N = 43
Descriptive categories	Reflection, communication and feedback as learning tools Pedagogical framework and overall structure Patients’ participation Student collaboration Participating in projects	Students’ perception of supervision Models and strategies of supervision Relationship student-supervisor and learning environment Supervisors’ perceptions of supervisors’ role	Authentic situations and contexts Factors facilitating and hindering learning Encounters and relationships Developing professional competence and identity	Student experience Design and implementation of IPL Student collaboration Learning and IPL	Student perception of different aspects Diverse clinical settings Social interactions Intercultural and international aspects
Subject areas	6. Specific learning outcomes *N* = 19	7. Co-operation university clinical setting *N* = 15	8. Relation to evidence-based knowledge *N* = 10	9. Patients’ role *N* = 6	
Descriptive categories	Professional characteristics/ capacity/skills Knowledge transfer	Collaboration based on models/framework Role of the clinical teacher Educational interventions and evaluation	Students applying/utilizing research Students participating in research projects	Patients’ experience Student’s experience Patient involvement in students learning	
Number of articles					Total: 391

#### Organisation of the clinical practice

The focus of the purposes and research questions in this subject area was on how the clinical practice was organised to support students’ learning. The five identified categories are pedagogical framework and overall structure; reflection, communication and feedback as learning tools; patient participation; student collaboration and participating in projects. Pedagogical framework was about applying learning theories or concepts and/or models of learning when organising the clinical practice. Within this category, task-based learning, model of social learning and collaborative learning were investigated. Learning tools, such as reflection, communication and feedback, were applied and evaluated to reach an understanding about how to gain professional development and competence. These tools were used both in discussions and in writing, and students received feedback not only from supervisors but also from peers and patients. Research about models concerning patients’ participation in students’ learning was based on patients being experts on their situation or as professional patients allowing students to practice procedures on them. Student collaborations were studied in relation to organising for students to work and learn in pairs as well as in groups in student run clinics/wards. The initiative to involve students in research projects was also studied and included in the categories within this subject area.

#### Supervision

The subject area *supervision* focused on research related to students’ perception of supervision; models and strategies of supervision; the student-supervisor relationship and learning environment.

Within the categories, students’ perceptions of how they were welcomed in clinical practice, how the supervision was organised and their relationship to their supervisors were investigated. The student-supervisor relationship included even supervisors’ perceptions of their role as supervisors supporting students’ learning. Different strategies of supervision and supervision models were also described. Interaction, feed-back, assessment, mentorship, process-oriented supervision, and group supervision were included in the categories.

#### Students’ experiences

The subject area *students’ experience* focused on students expressing what and how they learn in clinical practice. It consists of four categories: authentic contexts and situations, factors facilitating and hindering learning, encounters and relationships, and developing professional competence and identity. Authentic contexts and situations investigated students’ experience of their learning in different clinical contexts and in real situations. The importance of the authentic context has been researched specifically in the beginning and nearing the end of education. Factors that either facilitated or hindered students such as emotions, stress, responsibility, challenges, and support were highlighted issues. Encounters and relationships between students and supervisors, as well as between students and patients, were inquired into from the perspective of students. Research about developing professional competence and identity included the transition from student to professional, being in real life situations, being involved and allowed to be involved, and how a student’s personal approach affected learning.

#### Interprofessional learning (IPL)

*Interprofessional learning* as a subject area was examined in relation to four identified categories: design and implementation of IPL, students’ experience, student collaboration, and students’ learning. The category design and implementation contained questions about different types of IPL wards/clinics, how they implemented the interprofessional training and how it was evaluated by the students. Students’ experience was about how the students perceived the clinical practice in the IPL context including perceptions of their own and other professions. Collaboration between the various student groups was investigated as teamwork and performing tasks related to their own professions and to the joint responsibility for patient care and patient safety. Similarities and differences between the different health care student groups was also analysed. In the category students’ learning, peer-learning, interprofessional learning activities, learning outcomes and development of interprofessional competence were researched. Furthermore, emotions, readiness for IPL, impact on students’ self-efficacy, and professional identity were issues studied within the category students’ learning.

#### Learning environment

The subject area *learning environment* consists of four identified categories: student perception of different aspects, diverse clinical setting, social interactions, and intercultural and international aspects. Questions related to students’ perceptions contained an evaluation of the overall learning environment, namely how the actual learning environment had affected their learning, which was determined by assessing the quality of their clinical practice. Clinical settings’ diversity, like in different medical specialties in acute and primary care settings, was researched to understand these contextual aspects of the learning environment. Social interactions were investigated to discover how students perceived the workplace culture through involvement, belongingness and socialisation. Additionally, questions concerning the characteristics of the learning environment, including the role of the manager, formed the focus in some studies. The learning environment was also researched in relation to how intercultural and international aspects concerned both how international students perceived the learning environment and how studying abroad was relevant for intercultural competence and working in the home country.

#### Specific learning outcomes

Specific learning outcomes as a subject area are described by two identified categories: professional characteristics/capacity/skills and knowledge transfer. Research concerning specific learning outcomes linked to the professional characteristics/capacity consists of the development of empathy, moral competence, persistence, engagement and self-reflection. Research concerning specific outcomes linked to professional skills described professional tasks and learning activities in specific contexts and other skills that are required in patient care. Knowledge transfer refers to exploring students’ ability to apply knowledge in different contexts.

#### Co-operation between university and clinical setting

Three identified categories describe the subject area *co-operation between university and clinical setting*: collaboration based on models/framework, role of the clinical teacher, and educational interventions and evaluation. Collaboration based on models/frameworks concerned shared involvement focusing on student learning and bridging for the joint task and responsibility. The role of the clinical teacher was related to questions about the presence of a clinical teacher in different settings and the meaning of a clinical teacher at different stages of students’ education. Educational interventions and follow-up concerned joint projects and how they have been evaluated.

#### Relation to evidence-based knowledge

The subject area *relation to evidence-based knowledge* focused on two identified categories: students participating in research projects and students applying/utilising research. In the category *students participating in research projects*, the questions concerned the impact on students learning in relation to collaboration and partnership between universities and clinical settings and how students were involved in research projects. *Students applying/utilising research* was about how students learn to practice evidence-based knowledge and their experiences of implementation.

#### Patients’ role

The subject area *patients’ role* consists of three identified categories: patients’ experience, students’ experience and patient involvement in students learning. Patients’ and students’ experience pictured their encounters and relationship, what happened and how it was expressed by both patients and students. Patient involvement in students’ learning was about in what ways patients were involved. Further, how the patients perceived being cared for by students and how the students perceived their learning from/with patients was also explored.

### Health care student groups

The number of articles per health care student group in each subject area is presented in Table 3. Nursing students was the most researched student group (74%) followed by medical students (20%). Articles including occupational therapist were 9% and physiotherapist students 8% and most of them were for both the professions in the subject area interprofessional learning. Only three articles included biomedical analyst students (1%), two of them in the subject area *interprofessional learning*. In 19 of the articles, it was not specified which health care programme the students belonged to; some of these articles included several professions.

**Table 3.** The number of articles per health care student group in each subject area.

	Organization of clinical practice	Supervision	Students’ experience	Inter-professional learning	Learning environment	Specific learning outcomes	Cooperation university- clinical setting	Relation to evidence-based knowledge	Patients’ role	Summary
Nursing students, undergraduate and postgraduate education	66	62	49	39	33	17	12	7	4	**N = 290** **(74%)**
Medical students	21	7	15	24	8	1	1	0	1	**N = 78 (20%)**
Physiotherapist students	4	5	2	17	1	0	0	2	1	**N = 32 (8%)**
Occupational therapist students	6	2	2	21	0	0	2	1	1	**N = 35 (9%)**
Biomedical analyst students	0	0	0	2	0	1	0	0	0	**N = 3 (1%)**
Health care students, not specified	3	2	3	8	3	0	0	0	0	**N = 19 (5%)**
**Summary *N* = 391**	**N = 100**	**N = 80**	**N = 71**	**N = 47**	**N = 43**	**N = 19**	**N = 15**	**N = 10**	**N = 6**	

Note: Total articles: 391.

Some articles contain more than one student group. The student group *Nursing students, first and second cycle* includes undergraduate nursing students, specialist nursing students, master students, radiography students and midwifery students. In the student group *Health care students, not specified*, it was not clear which health care program the students belonged to.

### Methodological approaches

The number of articles in each category of methodological approaches is presented in Table 4. The category *qualitative studies* included different approaches, sometimes only termed qualitative and in other cases termed phenomenology, interpretative, ethnography and different kinds of content analysis. Data collection methods varied and comprised individual and group interviews, questionnaires with open questions and answers, observations, logbooks, reflection journals and critical incidents. Studies using quantitative approaches were mainly based on questionnaires. The character of the questionnaires varied from validated instruments to questionnaires developed especially for the purpose of evaluating a specific intervention. The category with a combination of methodological approaches used different data collection methods in their studies. The most common combination was a structured questionnaire analysed statistically and open questions and answers analysed with content analysis. In the category of *reviews*, different kinds of systematic reviews were used. In some reviews only quantitative studies were included and in others different approaches were reviewed and synthesised. The category *descriptions* was characterised by studies describing an intervention, sometimes building on a theoretical framework. In other studies, the basis for the intervention was not spelled out clearly. None of these studies presented a systematic evaluation of the intervention and that is why they are not categorised in any of the other methodological categories.

**Table 4.** Methodological approaches of the studies.

Subject	Organisation clinical practice *N* = 100	Supervision *N* = 80	Students experience *N* = 71	Interprofessional learning *N* = 47	Learning environment *N* = 43	Specific learning outcomes *N* = 19	Co-operation university – clinic *N* = 15	Evidence based knowledge *N* = 10	Patients’ role *N* = 6	Summary *N* = 391 (100%)
Qualitative	58	56	49	23	26	13	5	8	5	*N* = 243 (62%)
Quantitative	14	10	16	12	15	3	4	1	0	*N* = 75 (19%)
Combination qualitative and quantitative	11	9	2	6	0	0	2	0	0	*N* = 30 (8%)
Review	5	3	4	4	2	2	2	0	1	*N* = 23 (6%)
Description	11	2	0	2	0	1	2	0	0	*N* = 18 (5%)
Unclear	1	0	0	0	0	0	0	1	0	*N* = 2 (0,2%)

The methodologies employed in relation to the identified subject areas varied. Within the subject area *interprofessional learning and cooperation between university and clinic*, the methodological approaches were more varied than within other subject areas. The qualitative approaches were common in the subject area *learning environment*, but a proportional higher number of studies used quantitative approaches compared to other subject areas. Qualitative methodological approaches were most common in the articles about nursing students and articles about medical students were more often quantitative.

## Discussion

In this scoping review 391 articles about characteristics of research concerning students’ learning in clinical practice were included based on studies in a Nordic context. The result show that this is an extensive research area providing perspectives and aspects illuminating the complexity of clinical practice. Some subject areas turned out to be well covered while other areas were sparsely researched. The most researched subject areas were *organisation of the clinical practice, supervision* and *students’ experiences*, with 168 studies exploring these subject areas (Table 2). Studies about *interprofessional learning* (47) and different aspects related to the clinical practice concerning *learning environment* (43) are also relatively frequently represented. The research coming to the fore within these subject areas provides a comprehensive basis for further analysis of common factors that can facilitate and support student learning in clinical practice. The subject that is least researched is the *patient´s role* (6) in relation to students’ learning. This is an alarming discrepancy given the importance for students to learn through encounters with patients [13,27]. Does this mean that the patients’ role is not acknowledged as important in student learning or are methods to include patients lacking? Patients´ participation in their care has also increased in recent times. Possible explanations to the lack of research could be that exploring patients’ experiences is controlled by several rules and regulations, which are aimed at protecting patients’ rights and vulnerability. These rules and regulations might hinder research, but at the same time there is an urgent need to know more about how patients experience their role and involvement in students’ learning. Questions concerning the *cooperation between the* university and the *clinical setting* (15) are also less researched according to this review. Correspondence between theoretical and practical education has been identified as important in students’ development of their profession [5,9] - [10]. To get a better understanding of factors impacting students’ learning related to this kind of cooperation there is a need to further explore issues within this subject area. Other less researched subjects are *specific learning outcomes* (19) and *relation to evidence-based knowledge* (10). These subjects concern more specific issues, which might be a reason why they are not so common in the research field. In a subsequent analysis about common denominators impacting students’ learning it is important to consider the lack of research within these subject areas.

The health care student group that is by far most studied is nursing students. One explanation for this could be that, apart from the fact that nursing students are a large group within health care profession students, nursing students’ curricula include considerable time in clinical practice. The European recognition of professional qualifications states that a large proportion of the nursing programme’s hours must be clinical training [28]. Another reason may be that questions about, for example, organisation and competence regarding supervision and assessment in clinical practice have traditionally been considered important in the Nordic countries to develop nursing education [1,18,19,29]. In further development of knowledge about students learning in clinical practice it is important to pay attention to the rich research produced within nursing. It is also essential to consider specific attributes and contextual factors related to students within medicine, physical therapy, occupational therapy, and biomedicine that may differ from nursing education.

Overall, the qualitative approaches predominated the methodologies of the studies. However, a certain variation of the choice of methodology linked to the identified subject areas and health care students could be noted. Qualitative methodological approaches were most common in the articles about nursing students, which can be explained by a long tradition of using qualitative methods in the field of nursing. In the same way, the articles about medical students were more often quantitative, as is the long tradition in medicine. These differences must be included in appraisal of findings in further development.

To reach a deeper pedagogical perspective and understanding of the conducted research in this review, the descriptions of the included articles are related to the analysis of pedagogical research projects supported by Region Stockholm and Karolinska Institutet presented in the method section. The analysis of these projects resulted in identification of significant factors and concepts that influence students’ learning, namely meaningfulness, belongingness, authenticity, trust, and learning process. Meaningfulness, belongingness, authenticity, and trust are concepts that are linked to *students’ experience, supervision, learning environment and patients’ role*. Meaningful learning is about going beyond recognition and recalling or constructing knowledge only from experiences. It is a metacognitive process involving understanding, applying, analysing, evaluating and creating in knowledge construction. If this metacognitive process is to happen, learning also needs to make sense for the learner [30,31]. Belongingness can be explained as an individual sense of belonging that is created in interaction between an individual and the environment. It has been shown to be crucial for learning concerning engagement, performance, and well-being [32]. Further, learning in a clinical context is based on trust and authenticity. As a student, entering the variety and complexity of contexts within health care may create uncertainty and overwhelming expectations. Trust involves judgment, competence, willingness, integrity, and the capacity to perform specific tasks/interventions in a specific context under particular conditions [33–35]. Authentic learning is about being given the opportunity for professional development and identifying the professional role in real-life settings [12,36]. Experience of authenticity, in turn, has been shown as the core of student learning in clinical context and involves all above mentioned concepts: meaningfulness, belongingness and trust [12–14,36]. Authenticity comprises both external and internal interacting dimensions, where the external dimension is related to the fact that the learning situation is situated in the real clinical environment and thus concrete, visual, and tangible [13,14]. Students can use all their senses to process information and learn, and internal authenticity is connected to students’ experiences of their actions and decisions having an actual impact on the patient and clinical work. It also involves feeling like one is experiencing being a significant part of the health care team, having opportunities to form a relationship, and feeling a moral responsibility for the patient and her/his actions [13,14]. Learning process as a concept can be related to the *organisation of clinical practice, interprofessional learning, learning outcomes, co-operation between university and clinical setting* and *the relation to evidence-based knowledge*. All levels of the health care organisation will be involved both directly and indirectly, and the students will have many interactions with staff, patients, and other health care students. It can be organised in different ways to create conditions to inspire and support students’ learning and professional development. Examples of initiatives aiming to prioritise students’ clinical education are clinical education wards focused on certain student groups or interprofessional learning [16,37]. Accordingly, the concept of learning processes is complex and involves several dimensions that influence learning. Razack and Philibert [38], Gruppen [2] and Chan et al. [3] discuss the complexity and multi-dimensionality concerning physical environment and infrastructure, social interaction and institutional cultures. The complexity and multi-dimensionality can be linked to our results concerning learning process and dimensions that influence students’ learning, which are to a great extent formed and framed by university and clinical practice.

This scoping review approach [23–25] was chosen to investigate previous research and provide an overview of the evidence and knowledge gaps concerning students’ learning in clinical practice. The chosen method made it possible to acquire an overview of publications and describe characteristics in this extensive research area. It would be possible to build on the results and continue research to create a comprehensive pedagogical framework on how to promote healthcare students’ learning in clinical practice. Some limitations should be noted. The included articles are only analysed concerning aims and research questions. To accomplish a deeper understanding of conducted research the results of the studies should be analysed. In such an analysis it would be interesting to find out more about similarities and differences between health care student groups. Another consideration to take into account is that this study relates to the Nordic countries. This means that this study is limited and so far only contributes with some aspects of what is known about factors that impact healthcare students’ learning in clinical practice. In order to create a pedagogical framework valid also for a wider context, further research is required, including international studies.

## Conclusion

Overall this scoping review showed that health care students’ learning in clinical practice has largely been researched within the Nordic countries. The analysis of research questions that had been examined showed that some subject areas, namely *organisation of the clinical practice, supervision* and *students’ experience*, predominated the conducted studies. Other subject areas, *interprofessional learning* and *learning environment* were also quite well represented while the important subject areas *co-operation between university and clinical setting* and *patients’ role* were sparsely investigated. Relating the identified subject areas in the studies to central learning concepts revealed that there is a rich body of knowledge that can be built on to formulate a comprehensive pedagogical framework within the field. However, it is urgent that the identified subject areas being less represented in research, especially the role of the patient and cooperation between clinical placements and the university, are highlighted in continued research. Variations regarding the type of health care students that have been involved in the conducted research as well as employed methodological approaches are other factors that must be considered in further development creating a pedagogical framework.

## Supplementary Material

Supplemental Material

Supplemental Material

Supplemental Material

Supplemental Material

Supplemental Material

## Appendix

**Study protocol**: Analysis of published articles based on pedagogical research projects funded by the Regional Agreement on Medical Training and Clinical Research between Region Stockholm and Karolinska Institutet

Title, authors, journal

**Table:**

Which aspects and factors concerning students learning in clinical practice are researched?	Which health care students are included	Context	Methods	Results

**Table:**

Conclusions	Theoretical framework	Core concepts	Part of doctoral thesis

**Table:**

Comments – methodological quality, relevance for the project

## Appendix 2: Documentation of search strategies

University Library search consultation group

Date: Mars 2020

Topic/research question: Studenters lärande i klinisk praktik

Name of researcher(s):

Librarian(s):

Databases:

1. Medline(OVID)
2. SveMed+
3. CINAHL

Total number of hits:

- Before deduplication: 5,920
- After deduplication: 3,126

Comments:

### PRISMA 2009 Flow Diagram^1^

1. Medline(OVID)

**Table:**

Interface: OVID Date of Search: 31 mars 2020 Number of hits: 3,011	Field labels exp/= exploded MeSH term /= non exploded MeSH term .ti,ab,kf. = title, abstract and author keywords adjx = adjacent within x words, regardless of order * = truncation of word for alternate endings
1. Clinical Clerkship/ 2. Interdisciplinary Placement/ 3. Internship, Nonmedical/ 4. Preceptorship/ 5. exp Learning/ 6. Mentoring/ 7. Mentors/ 8. (clerkship* or clinical* or facilitator* or internship* or learn* or mentor* or placement* or preceptorship or supervis* or tutor*).ti,ab,kf. 9. or/1–8 10. exp Students/ 11. student*.ti,ab,kf. 12. or/10–11 13. exp “Scandinavian and Nordic Countries”/ 14. Denmark.af. 15. Finland.af. 16. Greenland.af. 17. Iceland.af. 18. Norway.af. 19. Sweden.af. 20. or/13–19 21. 9 and 12 and 20 22. limit 21 to yr=“2000 -Current” 23. limit 22 to (danish or english or finnish or norwegian or swedish)	

**Table:** 2. SveMed+

Interface: Date of Search: 31 mars 2020 Number of hits: 566	Field labels [mh] = MeSH-termer exp = exploded MeSH-term
1. Clinical Clerkship [mh] 2. Interdisciplinary Placement [mh] 3. Internship, Nonmedical [mh] 4. exp Learning [mh] 5. Mentoring [mh] 6. Mentors [mh] 7. Preceptorship [mh] 8. 1 or2 or 3 or 4 or 5 or 6 or 7 9. exp Students [mh] 10 8 and 9 11. Begränsning år: 2000–2019, Språk: dansk, engelsk, norsk, svensk	

**Table:** 3. CINAHL

Interface: Ebsco Date of Search: 31 mars 2020 Number of hits: 2,343	Field labels MH+ = exploded CINAHL Heading MH = non exploded CINAHL Heading TI = title AB = abstract Nx = adjacent within x words, regardless of order * = truncation of word for alternate endings
S1 (MH “Education, Clinical+”) S2 (MH “Internship and Residency”) S3 (MH “Learning+”) S4 (MH “Mentorship”) S5 (MH “Preceptorship”) S6 (MH “Student Placement”) S7 TI ((clerkship* or clinical* or facilitator* or internship* or learn* or mentor* or placement* or preceptorship or supervis* or tutor*)) OR AB ((clerkship* or clinical* or facilitator* or internship* or learn* or mentor* or placement* or preceptorship or supervis* or tutor*)) S8 S1 OR S2 OR S3 OR S4 OR S5 OR S6 OR S7 S9 (MH “Students, Health Occupations+”) S10 TI student* OR AB student* S11 S9 OR S10 S12 (MH “Scandinavia+”) S13 AF ((Denmark or Finland or Greenland or Iceland or Norway or Sweden)) OR TI ((Denmark or Finland or Greenland or Iceland or Norway or Sweden)) OR AB ((Denmark or Finland or Greenland or Iceland or Norway or Sweden)) S14 S12 OR S13 S15 S8 AND S11 AND S14 S16 Limiters - Peer Reviewed; Published Date: 20000101–20201231. Narrow by Language: - Swedish, Finnish, Danish, Norwegian, English	
