# Supplementary material for: Students’ learning in clinical practice – a scoping review of characteristics of research in the Nordic countries
Source: Med Educ Online. 2023 Nov 18;28(1):2279347. doi: 10.1080/10872981.2023.2279347 (PMC11078069; doi:10.1080/10872981.2023.2279347)
Supplement: Supplemental Material [file ZMEO_A_2279347_SM5327.docx]

Appendix 2: Documentation of search strategies

University Library search consultation group

Date: Mars 2020

Topic/research question: Studenters lärande i klinisk praktik

Name of researcher(s):

Librarian(s):

Databases:

1. Medline(OVID)
2. SveMed+
3. Cinahl

Total number of hits:

- Before deduplication: 5,920
- After deduplication: 3,126

Comments:

**PRISMA 2009 Flow Diagram^[[1]](#footnote-1)^**

Full-text articles excluded based on the exclusion criteria
(n = 597)

Studies included in the analysis based on the inclusion criteria
(n = 391)

**Records identified through database searching
(n = 5,920 )**

## Screening

## Included

## Eligibility

## Identification

Additional records identified through other sources
(n = 0 )

**Records after duplicates removed
(n = 3126 )**

Records screened
(n = 3126 )

Records excluded
(n = 2138)

Abstract assessed for eligibility
(n = 988 )

1. Medline(OVID)

| Interface: OVID  Date of Search: 31 mars 2020  Number of hits: 3,011 | Field labels   - exp/ = exploded MeSH term - / = non exploded MeSH term - .ti,ab,kf. = title, abstract and author keywords - adjx = adjacent within x words, regardless of order - * = truncation of word for alternate endings |
| --- | --- |
| 1. Clinical Clerkship/  2. Interdisciplinary Placement/  3. Internship, Nonmedical/  4. Preceptorship/  5. exp Learning/  6. Mentoring/  7. Mentors/  8. (clerkship* or clinical* or facilitator* or internship* or learn* or mentor* or placement* or preceptorship or supervis* or tutor*).ti,ab,kf.  9. or/1-8  10. exp Students/  11. student*.ti,ab,kf.  12. or/10-11  13. exp "Scandinavian and Nordic Countries"/  14. Denmark.af.  15. Finland.af.  16. Greenland.af.  17. Iceland.af.  18. Norway.af.  19. Sweden.af.  20. or/13-19  21. 9 and 12 and 20  22. limit 21 to yr="2000 -Current"  23. limit 22 to (danish or english or finnish or norwegian or swedish) | |

2. SveMed+

| Interface:  Date of Search: 31 mars 2020  Number of hits: 566 | Field labels   - [mh] = MeSH-termer - exp = exploded MeSH-term |
| --- | --- |
| 1. Clinical Clerkship [mh]  2. Interdisciplinary Placement [mh]  3. Internship, Nonmedical [mh]  4. exp Learning [mh]  5. Mentoring [mh]  6. Mentors [mh]  7. Preceptorship [mh]  8. 1 or2 or 3 or 4 or 5 or 6 or 7  9. exp Students [mh]  10 8 and 9  11. Begränsning år: 2000-2019, Språk: dansk, engelsk, norsk, svensk | |

3. Cinahl

| Interface: Ebsco  Date of Search: 31 mars 2020  Number of hits: 2,343 | Field labels   - MH+ = exploded Cinahl Heading - MH = non exploded Cinahl Heading - TI = title - AB = abstract - Nx = adjacent within x words, regardless of order - * = truncation of word for alternate endings |
| --- | --- |
| S1 (MH "Education, Clinical+") S2 (MH "Internship and Residency") S3 (MH "Learning+") S4 (MH "Mentorship") S5 (MH "Preceptorship")  S6 (MH "Student Placement") S7 TI ( (clerkship* or clinical* or facilitator* or internship* or learn* or mentor* or placement* or preceptorship or supervis* or tutor*) ) OR AB ( (clerkship* or clinical* or facilitator* or internship* or learn* or mentor* or placement* or preceptorship or supervis* or tutor*) ) S8 S1 OR S2 OR S3 OR S4 OR S5 OR S6 OR S7  S9 (MH "Students, Health Occupations+") S10 TI student* OR AB student* S11 S9 OR S10  S12 (MH "Scandinavia+") S13 AF ( (Denmark or Finland or Greenland or Iceland or Norway or Sweden) ) OR TI ( (Denmark or Finland or Greenland or Iceland or Norway or Sweden) ) OR AB ( (Denmark or Finland or Greenland or Iceland or Norway or Sweden) ) S14 S12 OR S13  S15 S8 AND S11 AND S14  S16 Limiters - Peer Reviewed; Published Date: 20000101-20201231. Narrow by Language: - Swedish,  Finnish, Danish, Norwegian, English | |
|  | |

1. From: Moher D, Liberati A, Tetzlaff J, Altman DG, The PRISMA Group (2009). Preferred Reporting Items for Systematic Reviews and Meta-Analyses: The PRISMA Statement. PLoS Med 6(6): e1000097. doi:10.1371/journal.pmed1000097. For more information, visit [www.prisma-statement.org](http://www.consort-statement.org/). [↑](#footnote-ref-1)
