# Supplementary material for: Students’ learning in clinical practice – a scoping review of characteristics of research in the Nordic countries
Source: Med Educ Online. 2023 Nov 18;28(1):2279347. doi: 10.1080/10872981.2023.2279347 (PMC11078069; doi:10.1080/10872981.2023.2279347)
Supplement: Supplemental Material [file ZMEO_A_2279347_SM5321.docx]

**Appendix 1**

**Study protocol:** Analysis of published articles based on pedagogical research projects funded by the university and the local council for health care (reformulated to not be traceable).

Title, authors, journal

| Which aspects and factors concerning students learning in clinical practice are researched? | Which health care students are included | Context | Methods | Results |
| --- | --- | --- | --- | --- |
|  |  |  |  |  |

| Conclusions | Theoretical framework | Core concepts | Part of doctoral thesis |
| --- | --- | --- | --- |
|  |  |  |  |

| Comments – methodological quality, relevance for the project |
| --- |
|  |
